# Association between movement behavior patterns and cardiovascular risk among Chinese adults aged 40–75: a sex-specific latent class analysis

**DOI:** 10.1186/s12889-024-18573-z

**Published:** 2024-04-25

**Authors:** Yichao Chen, Yingqian Song, Nan Zhou, Weiwei Wang, Xin Hong

**Affiliations:** 1 Nanjing Medical University Affiliated Nanjing Center for Disease Control and Prevention, No. 2 Zi’ZhuLin, 210003 Nanjing, China; 2 Department of Epidemiology and Biostatistics, School of Public Health, Nanjing Medical University, Nanjing, China

**Keywords:** Latent class analysis, Sleep quality, Physical activity, Sedentary behavior, Cardiovascular diseases

## Abstract

**Background:**

Cardiovascular disease (CVD) is a major global health threat, particularly in China, contributing to over 40% of deaths. While sleep behaviors, sedentary behaviors, and physical activities are recognized as independent lifestyle risk factors for CVD, there remains limited understanding of specific movement behavior patterns and their CVD risks, especially considering sex-specific differences. This study examines movement behavior patterns among Chinese adults (40–75) and their associations with cardiovascular risk, with a focus on sleep, physical activity (PA), and sedentary behavior (SB).

**Methods:**

Data pertaining to 13,465 male participants and 15,613 female participants, collected from the Chronic Disease and Risk Factor Surveillance Survey in Nanjing from February 2020 to December 2022. The latent class analysis method was employed to identify underlying movement patterns across sexes. Multinomial logistic regression models assessed CVD risk, and the China-PAR model calculated 10-year risk.

**Results:**

Three male and four female movement patterns emerged. Active Movers (17.10% males, 5.93% females) adhered to PA recommendations but had poorer sleep quality. Moderate Achievers (61.42% males, 45.32% females) demonstrated moderate behavior. Sedentary Sleepers (21.48% males, 10.20% females) exhibited minimal PA but good sleep. Female Moderate Physical Activity (MPA) Dominant Movers demonstrated a prevalent adherence to recommended MPA levels. Active movers had the lowest CVD risk. After adjusting for potential confounders, moderate achievers (OR = 1.462, 95% CI 1.212, 1.764) and sedentary sleepers (OR = 1.504, 95% CI 1.211, 1.868) were both identified as being associated with a high-risk of cardiovascular diseases (CVDs) compared to active movers in males, demonstrating a similar trend for intermediate risk. Such associations were not statistically significant among females.

**Conclusions:**

Our study revealed sex-specific movement patterns associated with CVD risks among middle-aged Chinese adults. We suggest that adopting an active movement behavior pattern, characterized by meeting or exceeding recommended levels of vigorous physical activity (VPA) and reducing sedentary behavior, is beneficial for all middle-aged adults, particularly males. An active lifestyle could help counteract the adverse effects of relatively poor sleep quality on the risk of developing CVD in this population. Integrating sleep, PA, and SB information provides a holistic framework for understanding and mitigating CVD risks.

**Supplementary Information:**

The online version contains supplementary material available at 10.1186/s12889-024-18573-z.

## Introduction

Cardiovascular disease (CVD) poses a substantial global health threat, contributing significantly to mortality rates, with over 40% of deaths in China attributed to its prevalence [1]. Atherosclerotic cardiovascular disease (ASCVD), primarily comprising ischemic heart disease and ischemic stroke, has witnessed a noteworthy increase in mortality in recent decades [2]. In 2019, the prevalence of CVD in China was 120 million, representing a 140.02% increase since 1990 [3]. Mortality rates and the number of deaths related to ASCVD were notably higher among males compared to females in all stroke subcategories [4]. The escalating challenge of preventing and treating these conditions underscores the urgency for effective interventions [2].

Lifestyle intervention is crucial for preventing cardiovascular diseases, with sleep and physical activity (PA) ranking just below smoking as the most significant modifiable lifestyle factors, particularly in the middle-aged population [5]. Previous research suggests that maintaining a balanced moderate physical activity (MPA) and vigorous physical activity (VPA) reduce CVD mortality risk, while surpassing recommended levels (MPA ≥ 150 min per week or VPA ≥ 75 min per week) enhances the protective effect [6]. Conversely, sleep duration less than 6 h per day and sedentary duration more than 10 h are linked to higher CVD risk [7, 8]. Moreover, in terms of cardiovascular health, sleep, PA and sedentary behavior (SB) are closely intertwined [2, 9]. Poorer sleep quality and increased sedentary time have been linked to heightened CVD risk [10–12], particularly when combined with other modifiable risk factors such as smoking and excessive alcohol consumption, which may further elevate the risk of stroke and coronary heart disease (CHD) [13, 14]. Despite these findings, there is limited knowledge regarding the subgroup of underlying behavior patterns that allocate time to these different activities and their specific risk for cardiovascular diseases (CVDs). Furthermore, it is noteworthy that males and females may exhibit distinctly different behavior patterns, both in the behaviors themselves and in the resulting cardiovascular outcomes [15, 16].

Latent Class Analysis (LCA), acknowledged as a ‘person-oriented analysis,’ proficiently captures the synergistic effects of multiple factors [17]. This analytical approach categorizes observed variables using an unobserved latent categorical variable, effectively minimizing confounding. The identification of qualitatively distinguishable subgroups provides a nuanced understanding, valuable for examining treatment and prevention effects [18].

Given the importance of cardiovascular risk assessment, particularly in adults aged 40 to 75 suggested by ACC/AHA Guideline [9], our aim is to conduct analysis within this population and stratify it by sex. Our objectives were threefold: (1) identify qualitatively distinguishable movement patterns based on PA, SB, and sleep in Chinese adults aged 40–75; (2) explore the associations between the identified patterns and cardiovascular risk; and (3) assess whether these associations are consistent across male and female subsamples.

## Methods

### Study population

The population was comprised of participants from a survey of Chronic Disease and Risk Factor Surveillance in Nanjing, the capital of Jiangsu Province in China, which was conducted from February 2020 to December 2022. The study recruited subjects from 35 towns through a multi-stage sampling approach combining probability-proportional-to-size sampling and random sampling. The general information (i.e., residence, age, sex, educational level, and marital status, etc.) was collected through a standardized questionnaire validated based on the China Chronic Disease and Risk Factor Surveillance [19]. The response rate was 90.6%.

Participants were eligible if they: (a) had lived in Nanjing for at least one and a half years; (b)were willing to comply with the study protocol; (c) were between 40 and 75 years old. A total of 31,647 individuals were recruited for participation. Exclusions included 61 participants with incomplete data for calculating 10-Year ASCVD Risk, 2,050 participants with pre-existing CVDs, and 458 with cancer or other severe comorbidities. Finally, 13,465 male participants and 15,613 female participants were enrolled in this study.

### Measures of movement behaviors

The long version of the International Physical Activity Questionnaire (IPAQ) was utilized to gather information on accumulated Moderate Physical Activity (MPA) and Vigorous Physical Activity (VPA) per week. Physical activities encompassed all tasks related to employment or schooling, as well as transportation, household chores or maintenance, and recreational, sports, or leisure-time activities [20]. Following the recommendations for Exercise and Physical Activity refers to ASCVD risk [2, 9], MPA was classified into three levels: No MPA, Low MPA(< 150 min/week), and Optimal MPA(≥ 150 min/week). Similarly, VPA was categorized into three levels: No VPA, Low VPA(< 75 min/week), and Optimal VPA(≥ 75 min/week). SB was added by (1) average amount of time spent sitting, reclining, or lying down watching TV per day; (2) average amount of time spent sitting, reclining, or lying down using computer per day; (3) average amount of time spent sitting, reclining, or lying down using phone per day; and (4) average amount of time spent sitting, reclining, or lying down reading print products per day. Sleep quality was assessed by the Pittsburgh Sleep Quality Index (PSQI). PSQI consists of 19 self-reported items, encompassing seven aspects of sleep problems. The sum of scores across the seven components yields the global PSQI score (ranging from 0 to 21). A higher total PSQI score indicates poorer sleep quality [21]. Participants were classified into quartiles for sedentary behavior and sleep quality within male and female samples.

### Cardiovascular health assessment

We employed the China-PAR risk assessment model to predict the 10-year risk of atherosclerotic cardiovascular disease (ASCVD) within the Chinese population. Detailed information on the model has been elucidated elsewhere [22]. This comprehensive model incorporates several key factors, including gender, age, place of residence (urban or rural), regional location (north or south, demarcated by the Yangtze River), waist circumference (WC), total cholesterol (TC), high-density lipoprotein cholesterol (HDL-C), current blood pressure, usage of antihypertensive medication, diabetes status, current smoking habits, and family history of CVD.

The risk of CVD is stratified into three categories: “low” for estimated 10-year risk less than 5%, “intermediate” for estimated 10-year risk ranging between 5% and 9.9%, and “high” for estimated 10-year risk exceeding 10% [22].

### Definitions of other involved variables

The demographic data and anthropometric measures, including weight, height, WC, and blood pressure, were measured by anthropometric investigators using unified brands and models instruments. Systolic blood pressure (SBP) and diastolic blood pressure (DBP) were measured with the patient in the sitting position two times before and after a 10-minute rest, using a digital sphygmomanometer (OMRON HBP 1300, OMRON Co., Ltd., China). The body mass index (BMI) was calculated as weight in kg divided by height in m^2^.

After 10-hour overnight fasting, 5 mL venous blood sample was drawn into the tube with coagulant by a research nurse following standard operating procedures, and then transported via the cold chain within 24 h for laboratory tests. Serum fasting blood glucose (FBG), glycated hemoglobin (HbA_1c_), TC, TG, HDL-C,and low-density lipoprotein cholesterol (LDL-C) were measured in the automatic Cobas® 6000 analyzer (Roche Diagnostics GmbH, Ibaraki-ken, Japan).

Smoking was divided into never smoker, former smoker, and current smoker. Drinking alcohol more than once per month over the past 12 months prior to the interview was defined as current drinking.

### Statistical analysis

The Kruskal-Walli’s test and Chi-squared test were performed to examine the differences between identified classes.

We used LCA (R poLCA packages, version 1.6.0.1) to assign all patients to non-overlapping clusters in a data-driven fashion [17]. Following practitioner’s guides [23, 24], the optimal number of latent classes was determined through a meticulous evaluation, considering key statistical metrics. Specifically, we employed Bayesian Information Criteria (BIC), sample size adjusted BIC (SABIC), and log-likelihood ratio test, where smaller values were deemed more favorable. Additionally, we assessed the entropy for classification quality, aiming for values within the range of 0 to 1, with higher values, preferably exceeding 0.6, indicating a satisfactory classification performance. This comprehensive approach provided a quantitative basis for selecting the number of latent classes, ensuring the avoidance of overfitting or underfitting in our analysis.

The impact of different movement patterns on the intermediate and high-risk of CVDs was analyzed through multinomial logistic regression. Model 1 remained unadjusted, while Model 2 was adjusted for educational level, drinking status, and tea consumption frequency. Given that age, blood pressure, smoking status, and other factors have been included in the calculation of the 10-year ASCVD risk, and considering that age closely correlates with patterns of physical activity, sedentary behavior, and sleep and CVD risk, Model 3 was further adjusted for age. In the models for movement behaviors and 10-Year ASCVD Risk, the movement pattern with the lowest impact on the outcome was chosen as the reference.

Data management and all statistical analyses were carried out using R version 4.2.0 (R Foundation for Statistical Computing, http://www.cran.r-project.org/). Analysis results were considered statistically significant at the two-sided 5% level.

## Results

### Characteristics of participants by sex

Among the 29,078 participants (comprising 46.31% men), the average age was 56.41 years for men and 56.25 years for women. Notably, males presented a higher proportion of individuals with higher education compared to females. Additionally, males exhibited a predilection for smoking, alcohol consumption, and frequent tea intake. Furthermore, they displayed elevated SBP and DBP. Detailed information is provided in Table 1. Male also exhibited higher CVD risk than female, both in intermediate risk (33.87% vs. 22.92%) and in high risk (14.14% vs. 7.53%). 6.6% and 38.3% of females met the recommendations for VPA and MPA respectively, while 18.2% and 20.7% of males met the recommendations for VPA and MPA. Supplementary Fig. 1 demonstrates the distribution of movement characteristics by sex.

**Table 1:** Characteristics of participants by sex

Characteristics	Female	Male	*P*-value
**N (%)**	15,613 (53.69)	13,465 (46.31)	
**Age (years)**	56.25 (8.26)	56.41 (8.60)	0.103
**Education**			< 0.001
Primary school or below	8184 (52.42)	4077 (30.28)	
Middle school	6716 (43.02)	8377 (62.21)	
High school or above	713 (4.57)	1011 (7.51)	
**BMI (kg/m** ^**2**^ **)**	24.70 (3.43)	25.06 (3.30)	< 0.001
**WC (cm)**	82.50 (9.00)	87.73 (8.90)	< 0.001
**Smoking (%)**			< 0.001
Never smoker	15,452 (98.97)	5629 (41.80)	
Former smoker	29 (0.19)	1334 (9.91)	
Current smoker	132 (0.85)	6502 (48.29)	
**Alcohol drinking (%)**			< 0.001
No	14,530 (93.06)	7095 (52.69)	
Before 30 days	688 (4.41)	5107 (37.93)	
Within 30 days	395 (2.53)	1263 (9.38)	
**Tea consumption (%)**			< 0.001
No consumption	13,085 (83.81)	6783 (50.38)	
Sometimes consumption	269 (1.72)	346 (2.57)	
Frequent consumption	322 (2.06)	579 (4.30)	
Regular consumption	1937 (12.41)	5757 (42.76)	
**FBG (mmol/L)**	5.79 (1.49)	6.06 (1.79)	< 0.001
**HbA**_**1c**_ **(%)**	5.74 (0.83)	5.85 (1.00)	< 0.001
**TG (mmol/L)**	1.65 (1.20)	1.87 (1.73)	< 0.001
**TC (mmol/L)**	5.26 (1.05)	4.98 (1.02)	< 0.001
**HDL-C (mmol/L)**	1.51 (0.38)	1.37 (0.39)	< 0.001
**LDL-C (mmol/L)**	3.14 (0.86)	3.04 (0.84)	< 0.001
**SBP (mmHg)**	130.47 (19.08)	132.97 (17.69)	< 0.001
**DBP (mmHg)**	79.68 (10.27)	83.84 (10.88)	< 0.001
**Family history of CVD (%)**	3016 (19.32)	2023 (15.02)	< 0.001

Values are presented as mean ± standard deviation or number (%). Abbreviations: BMI, body mass index; WC, waist circumference; FBG, fasting blood glucose; TG, triglycerides; TC, total cholesterol; HDL-C, high-density lipoprotein cholesterol; LDL-C, low-density lipoprotein cholesterol; SBP, systolic blood pressure; DBP, diastolic blood pressure; CVD, cardiovascular disease

### Characteristics of identified movement behavior patterns

Fit indices, including BIC, SABIC, entropy, and log-likelihood ratio test, were employed to assess the model fit across six latent profile models within a final sample size of 13,465 male participants and 15,613 female participants. Evaluation of the fit indices indicated that, for male participants, the optimal solution was the 3-class model, while for female participants, the 4-class model demonstrated the best fit. Further details regarding model selection statistics can be found in Supplementary Figs. 2 and 3.

**Fig. 1:**
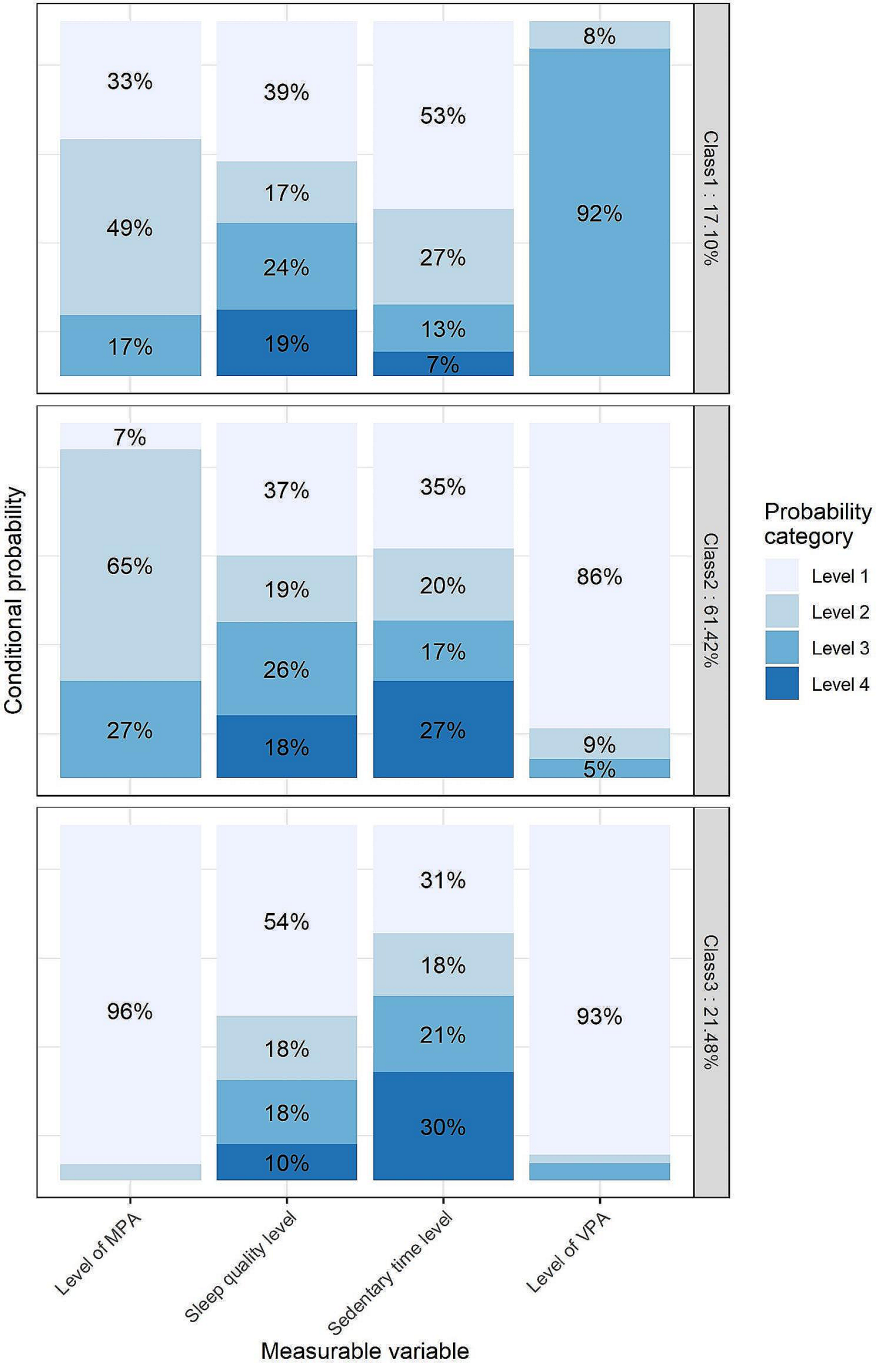
Conditional probabilities in a three latent Class model among male Participants. MPA: Moderate Physical Activity, categorized into three levels: 0, < 150 min, ≥ 150 min. Sleep Quality Level: Divided into four levels based on quartiles of the PSQI (Pittsburgh Sleep Quality Index). Sedentary Time Level: Categorized into four levels according to quartiles of daily sedentary time. VPA: Vigorous Physical Activity, divided into three levels: 0, < 75 min, ≥ 75 min. For ease of readability, conditional probabilities less than 5% are not displayed in the figure

**Fig. 2:**
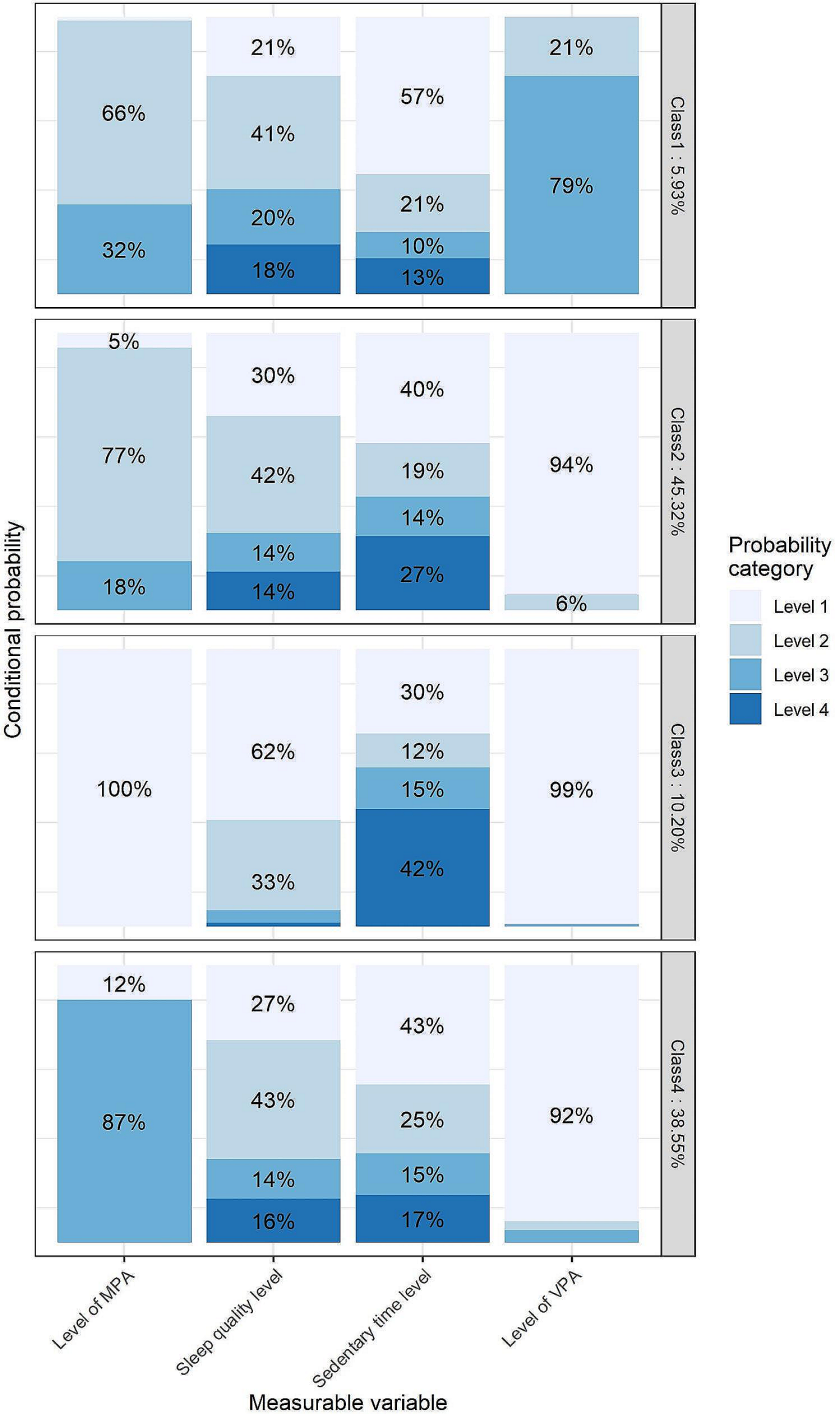
Conditional probabilities in a four latent Class model among female Participants. MPA: Moderate Physical Activity, categorized into three levels: 0, < 150 min, ≥ 150 min. Sleep Quality Level: Divided into four levels based on quartiles of the PSQI (Pittsburgh Sleep Quality Index). Sedentary Time Level: Categorized into four levels according to quartiles of daily sedentary time. VPA: Vigorous Physical Activity, divided into three levels: 0, < 75 min, ≥ 75 min. For ease of readability, conditional probabilities less than 5% are not displayed in the figure

For convenient reference, each class is identified by its lead or key behavioral trait, representing one or two conditions with the highest or lowest cluster-specific conditional probability, respectively. Three movement classes for males and four for females were identified, with male classes 1 to 3 sharing similar characteristics with female classes 1 to 3. According to the China-PAR risk assessment model, the predicted number of participants allocated to different classes at high risk for CVD over the next 10 years were as follows: 201 (8.73%), 1,272 (15.38%), and 431 (14.90%) for men, and 51 (5.36%), 559 (7.90%), 145 (9.11%), and 421 (6.99%) for women. Further details regarding risk across latent classes can be found in Supplementary Table 1. The results of the conditional probability distribution for each item of each sex are illustrated in Figs. 1 and 2.

In movement class 1, comprising 17.10% of male participants and 5.93% of female participants (named “Active Movers”), both sexes showcased optimal adherence to physical activity recommendations, with 92% and 79% exceeding 75 min of VPA, respectively. They exhibited the lowest sedentary behavior time (7% for males and 13% for females in quartile 4), yet showed relatively poor sleep quality, with the highest proportion in quartile 4 of the PSQI score (19% for males and 18% for females).

Male movement class 2 (61.42% of males, named “Moderate Achievers”) had 27% achieving MPA > 150 min, with 18% and 27% in quartile 4 of the PSQI score and sedentary time, respectively. Similarly, female movement class 2 (45.32% of females) exhibited comparable traits, with 18% achieving MPA > 150 min, and 14% and 27% in quartile 4 of the PSQI score and sedentary time, respectively.

Movement class 3, named “Sedentary Sleepers,” represented 21.48% of males and 10.20% of females. This class was characterized by minimal physical activity, with the highest proportion in quartile 4 of sedentary time (30% for males, 42% for females) and the lowest in quartile 4 of the PSQI score (10% for males, 0.1% for females).

Female class 4 was named the “MPA Dominant Movers”, with 87% of participants achieving MPA > 150 min.

The characteristics of classified participants were given in Tables 2 and 3. There were evident differences in education status, tea consumption frequency, fasting blood glucose, and family history of CVD in both sexes.

**Table 2:** Characteristics of male movement behavior patterns

Characteristics	Active Movers	Moderate Achievers	Sedentary Sleepers	*P*-value
**N (%)**	2303 (17.10)	8270 (61.42)	2892 (21.48)	
**Age (years)**	54.40 (7.40)	56.95 (8.73)	56.51 (8.88)	< 0.001
**Education**				< 0.001
Primary school or below	901 (39.12)	2280 (27.57)	896 (30.98)	
Middle school	1368 (59.40)	5203 (62.91)	1806 (62.45)	
High school or above	34 (1.48)	787 (9.52)	190 (6.57)	
**BMI (kg/m** ^**2**^ **)**	24.94 (3.21)	25.08 (3.32)	25.12 (3.29)	0.131
**WC (cm)**	86.62 (8.89)	87.87 (8.79)	88.23 (9.15)	< 0.001
**Smoking**				< 0.001
Never smoker	809 (35.13)	3333 (40.30)	1487 (51.42)	
Former smoker	194 (8.42)	930 (11.25)	210 (7.26)	
Current smoker	1300 (56.45)	4007 (48.45)	1195 (41.32)	
**Alcohol drinking**				< 0.001
No	1035 (44.94)	4212 (50.93)	1848 (63.90)	
Before 30 days	1029 (44.68)	3238 (39.15)	840 (29.05)	
Within 30 days	239 (10.38)	820 (9.92)	204 (7.05)	
**Tea consumption**				< 0.001
No consumption	1057 (45.90)	3924 (47.45)	1802 (62.31)	
Sometimes consumption	77 (3.34)	212 (2.56)	57 (1.97)	
Frequent consumption	104 (4.52)	348 (4.21)	127 (4.39)	
Regular consumption	1065 (46.24)	3786 (45.78)	906 (31.33)	
**FBG (mmol/L)**	5.84 (1.59)	6.10 (1.84)	6.10 (1.77)	< 0.001
**HbA**_**1c**_ **(%)**	5.84 (0.97)	5.86 (1.01)	5.83 (0.99)	0.443
**TG (mmol/L)**	1.72 (1.61)	1.89 (1.70)	1.95 (1.92)	< 0.001
**TC (mmol/L)**	4.97 (1.00)	4.96 (1.01)	5.04 (1.05)	0.002
**HDL-C (mmol/L)**	1.40 (0.36)	1.35 (0.39)	1.37 (0.40)	< 0.001
**LDL-C (mmol/L)**	3.06 (0.80)	3.03 (0.85)	3.04 (0.86)	0.479
**SBP (mmHg)**	132.10 (18.11)	132.83 (17.59)	134.06 (17.57)	< 0.001
**DBP (mmHg)**	83.16 (10.97)	83.82 (10.79)	84.45 (11.04)	< 0.001
**Family history of CVD**	347 (15.07)	1333 (16.12)	343 (11.86)	< 0.001

Values are presented as mean ± standard deviation or number (%). Abbreviations: BMI, body mass index; WC, waist circumference; FBG, fasting blood glucose; TG, triglycerides; TC, total cholesterol; HDL-C, high-density lipoprotein cholesterol; LDL-C, low-density lipoprotein cholesterol; SBP, systolic blood pressure; DBP, diastolic blood pressure; CVD, cardiovascular disease

**Table 3:** Characteristics of female movement behavior patterns

Characteristics	Active Movers	Moderate Achievers	Sedentary Sleepers	MPA Dominant movers	*P*-value
**N (%)**	926 (5.93)	7076 (45.32)	1592 (10.20)	6019 (38.55)	
**Age (years)**	55.24 (7.25)	56.25 (8.53)	56.58 (9.01)	56.32 (7.85)	0.001
**Education**					< 0.001
Primary school or below	610 (65.87)	3461 (48.91)	730 (45.85)	3383 (56.21)	
Middle school	299 (32.29)	3196 (45.17)	753 (47.30)	2468 (41.00)	
High school or above	17 (1.84)	419 (5.92)	109 (6.85)	168 (2.79)	
**BMI (kg/m** ^**2**^ **)**	25.09 (3.46)	24.67 (3.33)	24.61 (3.35)	24.69 (3.56)	0.004
**WC (cm)**	82.91 (9.18)	82.46 (8.98)	82.88 (9.11)	82.40 (8.97)	0.125
**Smoking (%)**					0.006
Never smoker	914 (98.70)	6992 (98.81)	1575 (98.93)	5971 (99.20)	
Former smoker	1 (0.11)	15 (0.21)	8 (0.50)	5 (0.08)	
Current smoker	11 (1.19)	69 (0.98)	9 (0.57)	43 (0.71)	
**Alcohol drinking (%)**					< 0.001
No	817 (88.23)	6546 (92.51)	1544 (96.98)	5623 (93.42)	
Before 30 days	72 (7.78)	336 (4.75)	30 (1.88)	250 (4.15)	
Within 30 days	37 (4.00)	194 (2.74)	18 (1.13)	146 (2.43)	
**Tea consumption (%)**					< 0.001
No consumption	773 (83.48)	5925 (83.73)	1442 (90.58)	4945 (82.16)	
Sometimes consumption	18 (1.94)	121 (1.71)	17 (1.07)	113 (1.88)	
Frequent consumption	32 (3.46)	150 (2.12)	20 (1.26)	120 (1.99)	
Regular consumption	103 (11.12)	880 (12.44)	113 (7.10)	841 (13.97)	
**FBG (mmol/L)**	5.64 (1.20)	5.82 (1.57)	5.87 (1.36)	5.75 (1.46)	< 0.001
**HbA**_**1c**_ **(%)**	5.73 (0.80)	5.73 (0.86)	5.67 (0.73)	5.77 (0.82)	< 0.001
**TG (mmol/L)**	1.62 (1.23)	1.68 (1.27)	1.60 (1.05)	1.62 (1.13)	0.005
**TC (mmol/L)**	5.13 (1.04)	5.25 (1.06)	5.33 (1.08)	5.27 (1.04)	< 0.001
**HDL-C (mmol/L)**	1.49 (0.33)	1.50 (0.40)	1.55 (0.44)	1.50 (0.34)	< 0.001
**LDL-C (mmol/L)**	3.14 (0.82)	3.13 (0.88)	3.05 (0.93)	3.17 (0.84)	< 0.001
**SBP (mmHg)**	130.26 (19.00)	130.00 (19.32)	131.09 (17.63)	130.88 (19.17)	0.032
**DBP (mmHg)**	79.73 (10.04)	79.71 (10.33)	80.44 (9.80)	79.44 (10.35)	0.007
**Family history of CVD**	204 (22.03)	1393 (19.69)	201 (12.63)	1218 (20.24)	< 0.001

Values are presented as mean ± standard deviation or number (%). Abbreviations: BMI, body mass index; WC, waist circumference; FBG, fasting blood glucose; TG, triglycerides; TC, total cholesterol; HDL-C, high-density lipoprotein cholesterol; LDL-C, low-density lipoprotein cholesterol; SBP, systolic blood pressure; DBP, diastolic blood pressure; CVD, cardiovascular disease

### Relationship between movement patterns and CVD risk

The associations between the latent class groups and CVD risk are presented in Table 4. In the male sample, moderate achievers exhibited the strongest association with both high 10-year ASCVD risk, with an odds ratio (OR) of 2.194 (95% CI: 1.868, 2.576), and intermediate 10-year ASCVD risk, with an OR of 1.507 (95% CI: 1.359, 1.671), compared to active movers. Sedentary sleepers also showed a positive association with both high 10-year ASCVD risk, with an OR of 2.026 (95% CI: 1.688, 2.431), and intermediate 10-year ASCVD risk, with an OR of 1.379 (95% CI: 1.220, 1.558), compared to active movers.

**Table 4:** Relationship between movement patterns and CVD risk

	Model 1		Model 2		Model 3	
	Intermediate-risk	High-risk	Intermediate-risk	High-risk	Intermediate-risk	High-risk
**Male latent class**						
**Class 1**	**Ref**		**Ref**		**Ref**	
**Class 2**						
*OR* (95%*CI*)	1.507 (1.359, 1.671)	2.194 (1.868, 2.576)	1.765 (1.587, 1.962)	2.782 (2.36, 3.28)	1.203 (1.066, 1.357)	1.462 (1.212, 1.764)
*P-*value	< 0.001	< 0.001	< 0.001	< 0.001	< 0.05	< 0.001
**Class 3**						
*OR* (95%*CI*)	1.379 (1.220, 1.558)	2.026 (1.688, 2.431)	1.585 (1.398, 1.797)	2.438 (2.021, 2.940)	1.225 (1.060, 1.415)	1.504 (1.211, 1.868)
*P-*value	< 0.001	< 0.001	< 0.001	< 0.001	< 0.05	< 0.001
**Female latent class**						
**Class 1**	***Ref***		***Ref***		***Ref***	
**Class 2**						
*OR* (95%*CI*)	1.085 (0.919, 1.279)	1.541 (1.144, 2.075)	1.29 (1.09, 1.527)	1.942 (1.437, 2.624)	0.941 (0.768, 1.152)	1.045 (0.734, 1.488)
*P-*value	0.336	< 0.05	< 0.05	< 0.001	0.555	0.806
**Class 3**						
*OR* (95%*CI*)	1.181 (0.972, 1.435)	1.844 (1.322, 2.573)	1.472 (1.206, 1.796)	2.507 (1.787, 3.516)	0.954 (0.748, 1.218)	1.151 (0.768, 1.726)
*P-*value	0.093	< 0.001	< 0.001	< 0.001	0.705	0.496
**Class 4**						
*OR* (95%*CI*)	1.127 (0.954, 1.332)	1.375 (1.017, 1.858)	1.226 (1.035, 1.452)	1.531 (1.129, 2.077)	0.983 (0.803, 1.205)	1.024 (0.717, 1.461)
*P-*value	0.159	< 0.05	< 0.05	< 0.05	0.872	0.898

China-PAR predicted the 10-year ASCVD risk across different movement patterns. Multinomial logistic regression was analyzed for the association between movement patterns and CVDs in male and female participants. Model 1 was unadjusted. Model 2 was adjusted for education, drinking, and tea consumption frequency. Model 3 was adjusted for education, drinking, tea consumption frequency and age

In the female sample, moderate achievers (OR = 1.541; 95% CI: 1.144–2.075), sedentary sleepers (OR = 1.844; 95% CI: 1.322–2.573), and MPA Dominant movers (OR = 1.375; 95% CI: 1.017–1.858) were associated with an increased high 10-year ASCVD risk but not the intermediate risk compared to active movers. All associations with CVDs became stronger after adjustment for potential confounders, including educational level, drinking status, and tea consumption frequency in Model 2.

All associations with CVDs become stronger after adjusted to potential confounders, educational level, drinking status, and tea consumption frequency in model 2. After further adjustment for age (model 3), such association disappeared in female sample but persisted in the male sample. Sedentary sleepers had the strongest association with both high 10-year ASCVD risk, with an OR of 1.504 (95% CI: 1.211, 1.868), and intermediate 10-year ASCVD risk, with an OR of 1.225 (95% CI: 1.060, 1.415), compared to active movers. Moderate achievers exhibited an OR of 1.462 (95% CI: 1.212, 1.764) associated with high 10-year ASCVD risk and an OR of 1.203 (95% CI: 1.066, 1.357) associated with intermediate 10-year ASCVD risk compared to active movers.

## Discussion

### Summary of findings

This study provides further evidence of the existence of movement patterns by sex. Beyond this, we provide new evidence on their clinical relevance, demonstrating that movement patterns are associated with differential risk for CVD among different sexes aged 40–75. We classified movement patterns into three latent classes for male (active movers, moderate achievers, and sedentary sleepers) and four latent classes, one unique for female (active movers, moderate achievers, sedentary sleepers, and MPA dominant mover). Active movers exhibited lowest CVD risk, while moderate achievers and sedentary sleepers exhibited differentially elevated CVD risk in different sexes. To the best of our knowledge, this study is among the first to investigate associations between CVDs and movement patterns (sleep, PA, and SB) using a person-oriented analysis method with representative data.

### Movement behavior patterns

Over the past decade, a global surge in sedentary lifestyles has led to adverse health impacts, including heightened all-cause mortality and cardiovascular diseases [25, 26]. The full 24-h cycle comprises four main movement behaviors: sleep, sedentary behavior (SB), light-intensity physical activity (LPA), and MVPA [9]. Previous evidence suggested that altering daily movement behavior compositions to incorporate more MVPA while reducing any other movement behavior, could help to improve cardiometabolic health in middle adulthood [27]. Active movers, characterized by high VPA and MPA but lower sleep quality, exhibited the lowest CVD risk. Consistent with prior research, higher PA or recommended moderate to vigorous physical activity (MVPA) mitigated the detrimental effects of short or long sleep duration on CVD mortality risks [28]. Moderate Achievers, constituting a significant proportion of the underlying patterns, demonstrated that while sleep quality remained unchanged or slightly improved, a substantial decrease in physical activity may contribute to an increased risk of serious CVD [12]. Sedentary Sleepers, characterized by extremely low physical activity and prolonged sedentary time, exhibited a high CVD risk. Our study builds upon previous findings and expands the understanding of associations between cardiovascular health and movement patterns across three dimensions [9]. This surpasses conventional focuses on individual or dual dimensions. Besides, several studies to date offer convincing evidence that similar inactive movement patterns are associated with a higher likelihood of obesity and worse cardio-metabolic health markers [29, 30].

### Sex differences

Ample evidence indicates that due to various societal factors, such as working duty and income disparities, women have been shown to experience a higher prevalence of all types of sleep disturbances compared to men [31], and women tend to exhibit different PA behaviors compared to men, influenced by factors such as external motives related to body image and appearance [32]. Previous study in Japanese adults has found that each 60 min unit of SB replaced with MPA or VPA was favorably associated with better sleep quality only among middle-aged women [33]. In this study, the constitution of underlying pattern differed across sexes. Notably, the proportion of active movers differed significantly between genders, with 17.01% of males compared to 5.93% of females falling into this category. This disparity exceeds previous findings reported in the Canadian population [34]. Male moderate achievers exhibited poorer sleep quality, contributing to a notably higher risk of CVDs compared to their female counterparts. Additionally, female sedentary sleepers displayed prolonged sedentary behavior and lower levels of physical activity, associated with an elevated risk of CVDs. These findings align with prior research indicating that sedentary behaviors may have more adverse health effects in females than males [34].

### Implications

Despite variations in included variables across studies in the context of CVD health, Sitting time has been associated with CVD mortality outcomes, showing a nearly dose-response relationship, particularly notable in individuals with low PA levels (reporting less than 150 min of MVPA per week) [35]. The introduction of the sleep quality dimension in our study addresses a key limitation by moving beyond the isolation of health impacts attributed to one or two movement behaviors. Furthermore, our approach integrates data and consensus to better elucidate the heterogeneity within a large sample of individuals of a specific age group, who are recommended to undergo routine assessments for traditional CVD risk factors [9]. A unique aspect of our study, building upon prior research in this field, involves the consideration of the lowest end of the intensity continuum. Specifically, our findings highlight that sedentary sleepers in male, characterized by extremely low physical activity and high sedentary behavior, exhibit a correlation with elevated cardiovascular diseases. This marks a progression beyond the scope of previous studies on sedentary behavior and cardiovascular diseases [10]. For individuals accustomed to a sedentary sleep pattern, initiating physical activities at low intensity for short durations and gradually increasing is recommended [2].Lastly, those accustomed to a moderate achiever pattern should consistently and incrementally increase their activity levels according to their abilities to minimize the risk of cardiovascular diseases.

### Limitations

This study has limitations. Firstly, at this stage, we refrain from attributing distinct patterns to varied CVD etiologies, acknowledging that our grouping may not optimally represent underlying movement patterns. Additionally, the cross-sectional nature of our study precludes making causal inferences from the results. The potential for patients to transition between classes should be explored in future investigations.

Furthermore, questionnaire surveys, including the International Physical Activity Questionnaire (IPAQ) and Pittsburgh Sleep Quality Index (PSQI), were utilized to gather information on physical activity and sleep quality, which may potentially lead to recall bias. Both questionnaires have been previously validated in the Chinese population [36, 37].

Moreover, emerging research and guidelines suggest that light-intensity physical activity, as a component of the 24-hour cycle, may play a crucial role in promoting health [9, 38]. However, the quantification of light-intensity physical activity in our study was challenging due to the lack of an objective measure.

Lastly, our research focused on middle-aged Chinese adults, a population routinely recommended for cardiovascular disease risk evaluation. The applicability of our strategy to individuals of other ethnicities and younger age groups needs thorough assessment in future prospective studies.

## Conclusions

In conclusion, our study reveals distinct movement patterns associated with specific CVD risks among middle-aged adults, emphasizing the importance of sex-specific considerations. Comprehensive health education and targeted interventions are crucial for individuals with high-risk movement patterns. Active movement behavior pattern is recommended for all middle-aged adults, particularly males. The integration of information across sleep, PA, and SB dimensions surpasses the insights derived from a singular perspective, providing a holistic framework for personalized prevention and intervention in the context of cardiovascular and other chronic diseases. In addition, we recommend further exploration of advanced methodologies, including LCA and other artificial intelligence techniques, to enhance resolution and predictive power in future research.

### Electronic supplementary material

Below is the link to the electronic supplementary material.

Supplementary Material 1

## Data Availability

The datasetsare available from The Nanjing Chronic Disease and Risk Factor Surveillance (NCDRFS), but restrictions apply to the availability of these data, which were used under licence for the current study and so are not publicly available. The datasets used and/or analysed during the current study are available from the corresponding author on reasonable request.

## Abbreviations

CVD: Cardiovascular Disease
ASCVD: Atherosclerotic Cardiovascular Disease
PA: Physical Activity
SB: Sedentary Behavior
MPA: Moderate Physical Activity
VPA: Vigorous Physical Activity
MVPA: Moderate To Vigorous Physical Activity
CVDs: Cardiovascular Diseases
LCA: Latent Class Analysis
IPAQ: International Physical Activity Questionnaire
PSQI: Pittsburgh Sleep Quality Index
WC: Waist Circumference
TC: Total Cholesterol
HDL-C: High Density Lipoprotein Cholesterol
SBP: Systolic Blood Pressure
DBP: Diastolic Blood Pressure
BMI: Body Mass Index
FBG: Serum Fasting Blood Glucose
HbA1c: Glycated Hemoglobin
LDL-C: Low Density Lipoprotein Cholesterol
BIC: Bayesian Information Criteria
SABIC: Sample Size Adjusted BIC
OR: Odds Ratio

## References

[1] Zhou M, Wang H, Zeng X, Yin P, Zhu J, Chen W (2019). Mortality, morbidity, and risk factors in China and its provinces, 1990–2017: a systematic analysis for the global burden of Disease Study 2017. Lancet.

[2] Chinese Guideline on the Primary Prevention of Cardiovascular Diseases (2021). Cardiol Discovery.

[3] Zhang J, Tong H, Jiang L, Zhang Y, Hu J (2023). Trends and disparities in China’s cardiovascular disease burden from 1990 to 2019. Nutr Metab Cardiovasc Dis.

[4] Ma Q, Li R, Wang L, Yin P, Wang Y, Yan C (2021). Temporal trend and attributable risk factors of stroke burden in China, 1990–2019: an analysis for the global burden of Disease Study 2019. Lancet Public Health.

[5] Tian F, Chen L, Qian ZM, Xia H, Zhang Z, Zhang J (2023). Ranking age-specific modifiable risk factors for cardiovascular disease and mortality: evidence from a population-based longitudinal study. EClinicalMedicine.

[6] López-Bueno R, Ahmadi M, Stamatakis E, Yang L, Del Pozo Cruz B (2023). Prospective associations of different combinations of aerobic and muscle-strengthening activity with All-Cause, Cardiovascular, and Cancer Mortality. JAMA Intern Med.

[7] Lao XQ, Liu X, Deng HB, Chan TC, Ho KF, Wang F (2018). Sleep Quality, Sleep Duration, and the risk of Coronary Heart Disease: a prospective cohort study with 60,586 adults. J Clin Sleep Med.

[8] Pandey A, Salahuddin U, Garg S, Ayers C, Kulinski J, Anand V (2016). Continuous dose-response Association between Sedentary Time and Risk for Cardiovascular Disease: a Meta-analysis. JAMA Cardiol.

[9] Arnett DK, Blumenthal RS, Albert MA, Buroker AB, Goldberger ZD, Hahn EJ (2019). 2019 ACC/AHA Guideline on the primary Prevention of Cardiovascular Disease: executive Summary. J Am Coll Cardiol.

[10] Bellettiere J, LaMonte MJ, Evenson KR, Rillamas-Sun E, Kerr J, Lee IM (2019). Sedentary Behavior and Cardiovascular Disease in Older Women. Circulation.

[11] Zhou L, Yu K, Yang L, Wang H, Xiao Y, Qiu G et al. Sleep duration, midday napping, and sleep quality and incident stroke. Neurology. 2020;94(4). 10.1212/WNL.0000000000008739 31827003

[12] Huang B-H, Duncan MJ, Cistulli PA, Nassar N, Hamer M, Stamatakis E (2022). Sleep and physical activity in relation to all-cause, cardiovascular disease and cancer mortality risk. Br J Sports Med.

[13] O’Donnell MJ, Chin SL, Rangarajan S, Xavier D, Liu L, Zhang H (2016). Global and regional effects of potentially modifiable risk factors associated with acute stroke in 32 countries (INTERSTROKE): a case-control study. Lancet.

[14] Jackson SE, Brown J, Ussher M, Shahab L, Steptoe A, Smith L (2019). Combined health risks of cigarette smoking and low levels of physical activity: a prospective cohort study in England with 12-year follow-up. BMJ Open.

[15] O’Neil A, Scovelle AJ, Milner AJ, Kavanagh A (2018). Gender/Sex as a Social Determinant of Cardiovascular Risk. Circulation.

[16] Madsen TE, Samaei M, Pikula A, Yu AYX, Carcel C, Millsaps E (2022). Sex differences in physical activity and incident stroke: a systematic review. Clin Ther.

[17] Linzer DA, Lewis JB (2011). poLCA: an R package for polytomous variable latent class analysis. J Stat Softw.

[18] Lanza ST, Rhoades BL (2011). Latent class analysis: an alternative perspective on Subgroup Analysis in Prevention and Treatment. Prev Sci.

[19] Zhang M, Wang L, Wu J, Huang Z, Zhao Z, Zhang X (2022). Data Resource Profile: China Chronic Disease and risk factor surveillance (CCDRFS). Int J Epidemiol.

[20] Craig CL, Marshall AL, Sjöström M, Bauman AE, Booth ML, Ainsworth BE (2003). International physical activity questionnaire: 12-country reliability and validity. Med Sci Sports Exerc.

[21] Buysse DJ, Reynolds CF, Monk TH, Berman SR, Kupfer DJ (1989). The Pittsburgh Sleep Quality Index: a new instrument for psychiatric practice and research. Psychiatry Res.

[22] Yang X, Li J, Hu D, Chen J, Li Y, Huang J (2016). Predicting the 10-Year risks of atherosclerotic Cardiovascular Disease in Chinese Population. Circulation.

[23] Weller BE, Bowen NK, Faubert SJ (2020). Latent class analysis: a guide to best practice. J Black Psychol.

[24] Sinha P, Calfee CS, Delucchi KL (2020). Practitioner’s guide to latent class analysis: methodological considerations and common pitfalls. Crit Care Med.

[25] Yang L, Cao C, Kantor ED, Nguyen LH, Zheng X, Park Y et al. Trends in Sedentary Behavior among the US Population, 2001–2016. JAMA. 2019;321(16). 10.1001/jama.2019.3636 PMC6487546 31012934

[26] Park JH, Moon JH, Kim HJ, Kong MH, Oh YH (2020). Sedentary lifestyle: overview of updated evidence of potential health risks. Korean J Family Med.

[27] Farrahi V, Kangas M, Walmsley R, Niemelä M, Kiviniemi A, Puukka K (2021). Compositional associations of Sleep and activities within the 24-h cycle with Cardiometabolic Health Markers in adults. Med Sci Sports Exerc.

[28] Liang YY, Feng H, Chen Y, Jin X, Xue H, Zhou M (2023). Joint association of physical activity and sleep duration with risk of all-cause and cause-specific mortality: a population-based cohort study using accelerometry. Eur J Prev Cardiol.

[29] Kim Y, Barreira TV, Kang M (2016). Concurrent associations of physical activity and screen-based sedentary behavior on obesity among US adolescents: a latent class analysis. J Epidemiol.

[30] Devaney J, Chastin SFM, Palarea-Albaladejo J, Dontje ML, Skelton DA. Combined effects of Time spent in physical activity, sedentary behaviors and sleep on obesity and cardio-metabolic health markers: a Novel Compositional Data Analysis Approach. PLoS ONE. 2015;10(10). 10.1371/journal.pone.0139984 PMC4604082 26461112

[31] Alosta MR, Oweidat I, Alsadi M, Alsaraireh MM, Oleimat B, Othman EH (2024). Predictors and disturbances of sleep quality between men and women: results from a cross-sectional study in Jordan. BMC Psychiatry.

[32] Garg S (2023). Gender differences in pathways influencing leisure time physical activity: a structural equation analysis. Diabetes Metabolic Syndrome: Clin Res Reviews.

[33] Koohsari MJ, Yasunaga A, McCormack GR, Shibata A, Ishii K, Liao Y et al. Sedentary behaviour and sleep quality. Sci Rep. 2023;13(1). 10.1038/s41598-023-27882-z PMC9859796 36670182

[34] Kehler DS, Clara I, Hiebert B, Stammers AN, Hay JL, Schultz A et al. Sex-differences in relation to the association between patterns of physical activity and sedentary behavior with frailty. Arch Gerontol Geriatr. 2020;87. 10.1016/j.archger.2019.103972 31739110

[35] Lavie CJ, Ozemek C, Carbone S, Katzmarzyk PT, Blair SN (2019). Sedentary behavior, Exercise, and Cardiovascular Health. Circul Res.

[36] Yan D, Huang Y, Chen X, Wang M, Li J, Luo D. Application of the Chinese Version of the Pittsburgh Sleep Quality Index in people living with HIV: preliminary reliability and validity. Front Psychiatry. 2021;12. 10.3389/fpsyt.2021.676022 PMC8291081 34295273

[37] Deng HB, Macfarlane DJ, Thomas GN, Lao XQ, Jiang CQ, Cheng KK (2008). Reliability and validity of the IPAQ-Chinese: the Guangzhou Biobank Cohort study. Med Sci Sports Exerc.

[38] Blodgett JM, Ahmadi MN, Atkin AJ, Chastin S, Chan HW, Suorsa K et al. Device-measured physical activity and cardiometabolic health: the prospective physical activity, sitting, and Sleep (ProPASS) consortium. Eur Heart J. 2023. 10.1093/eurheartj/ehad717 PMC10849343 37950859

