# Supplementary material for: Association between movement behavior patterns and cardiovascular risk among Chinese adults aged 40–75: a sex-specific latent class analysis
Source: BMC Public Health. 2024 Apr 25;24:1170. doi: 10.1186/s12889-024-18573-z (PMC11047026; doi:10.1186/s12889-024-18573-z)
Supplement: Supplementary file 1 — Supplementary Material 1 [file 12889_2024_18573_MOESM1_ESM.docx]

**Supplementary materials**

**Supplementary Figure 1:** Jitter plot of movement characteristics by sex

**Supplementary Figure 2:** Model selection statistics for the latent class solution in male participants

**Supplementary Figure 3:** Model selection statistics for the latent class solution in female participants

**Supplementary Table 1:** CVD risk of the different latent classes in men and women

**
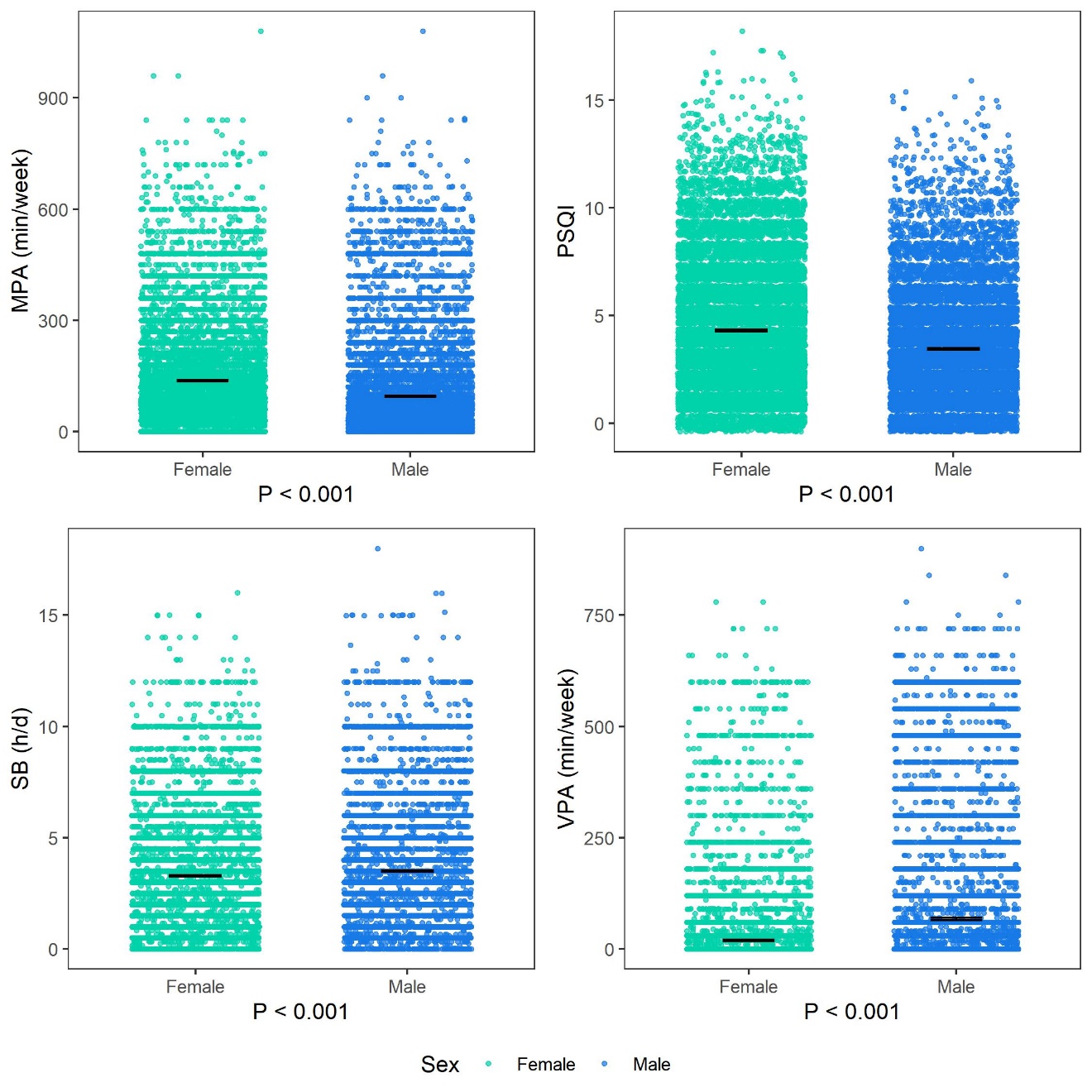
 Supplementary Figure 1**

**Jitter plot of movement characteristics by sex**

Figure legend:

*Abbreviations: MPA: moderate physical activity; PSQI: Pittsburgh Sleep Quality Index; SB: sedentary behavior; VPA: vigorous physical activity

Dashed line represents the mean value of movement characteristics by sex

**
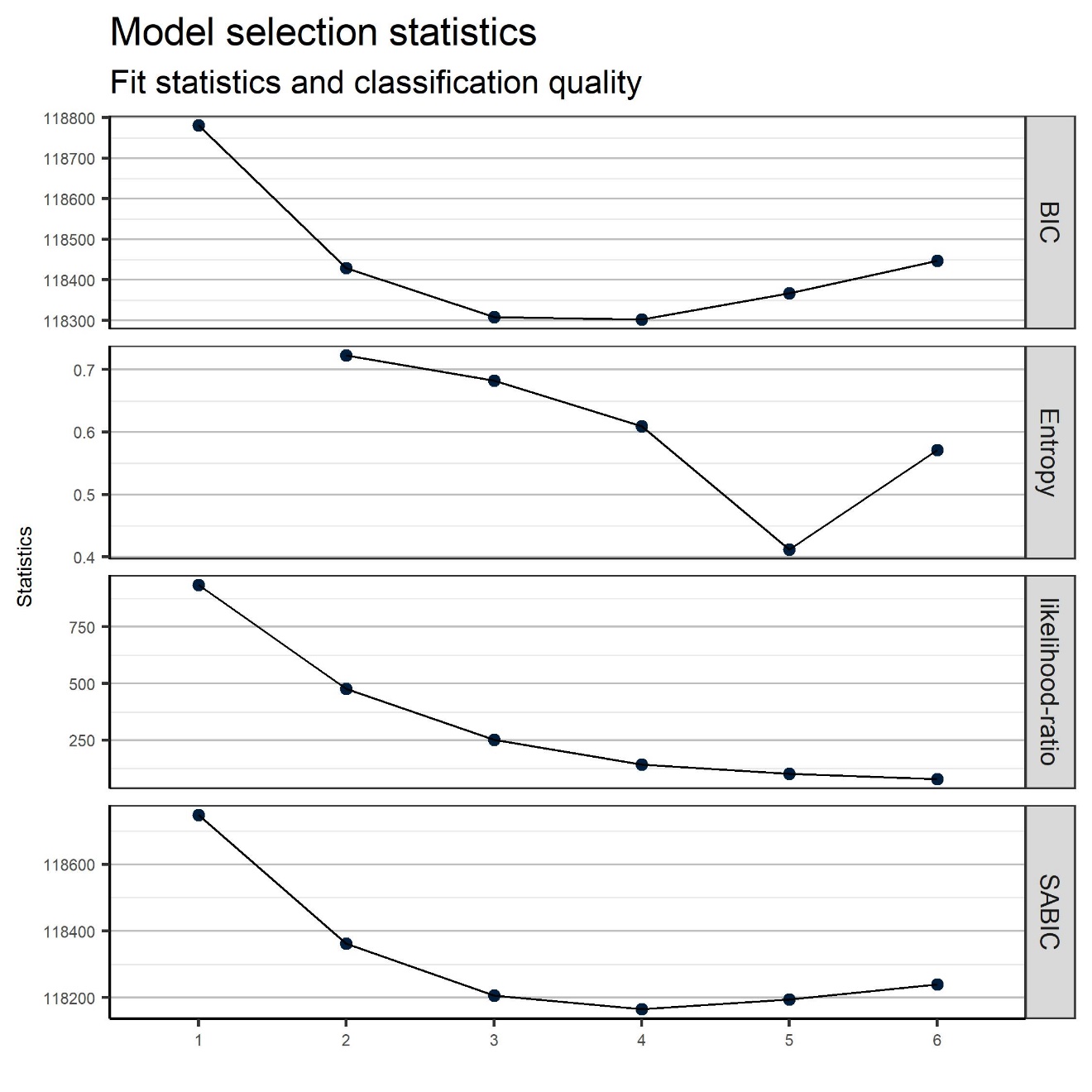
 Supplementary Figure 2**

**Model selection statistics for the latent class solution in male participants**

Best model: 3-class model.

Rationale: Candidates for the optimal model include the 3-class and 4-class models. However, introducing additional classes yields minimal improvement in class separation (with lower entropy), and both BIC and SABIC remain nearly unchanged compared to the 3-class model. Moreover, the 4-class model seems to compromise the clarity of clinical interpretation.
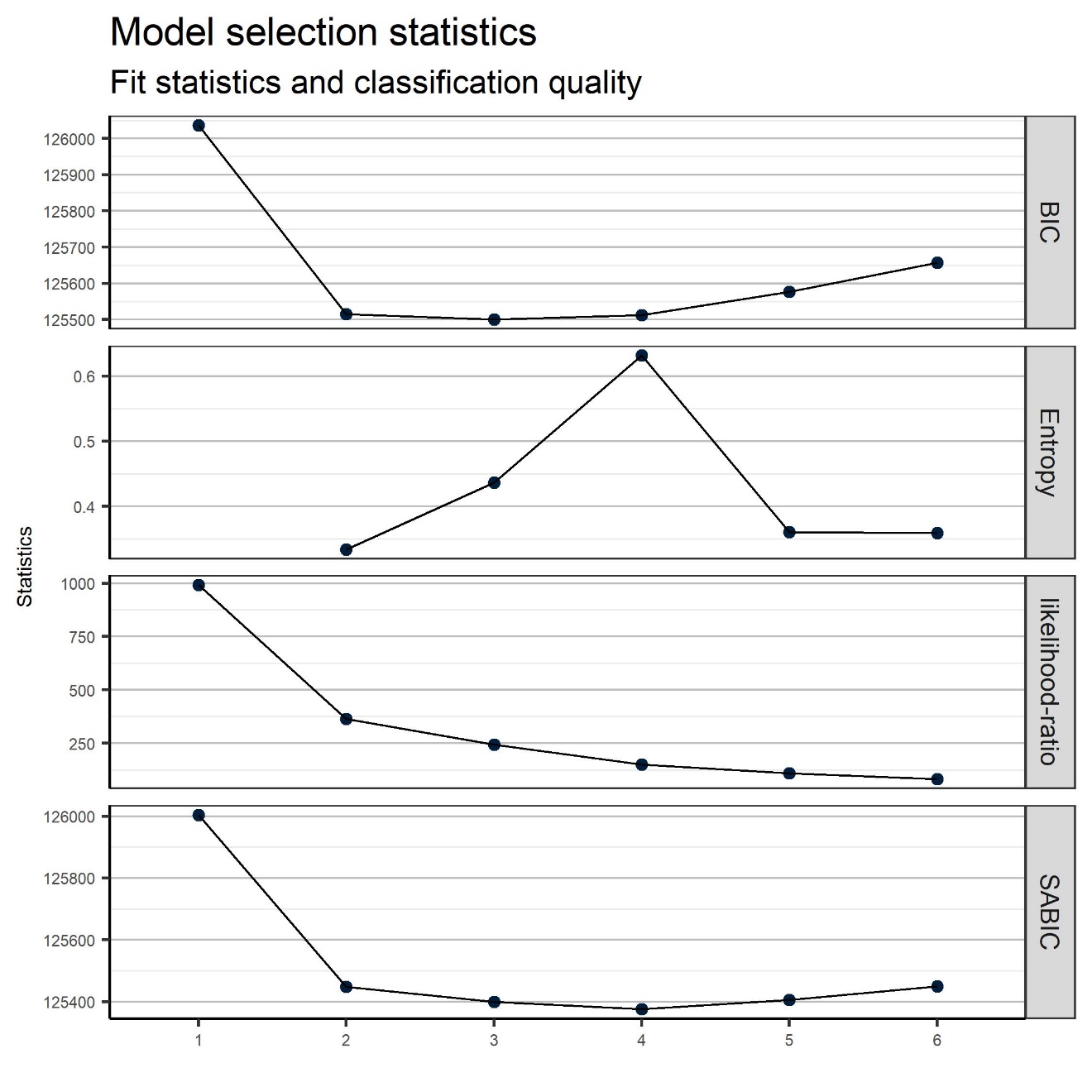
 **Supplementary Figure 3**

**Model selection statistics for the latent class solution in female participants**

Best model: 4-class model.

Rationale: The 4-class model exhibits the highest entropy among all classification models and demonstrates relatively low values for SABIC and BIC.

**Supplementary Table 1:** CVD risk of the different latent classes in men and women

| **Latent class** | **N (%)** | **Intermediate risk n(%)** | **High risk n (%)** |
| --- | --- | --- | --- |
| **Male** |  |  |  |
| Class 1 | 2303 (17.10) | 670(29.30) | 201 (8.73) |
| Class 2 | 8270 (61.42) | 2913(35.22) | 1272 (15.38) |
| Class 3 | 2892 (21.48) | 978(33.63) | 431 (14.90) |
| **Female** |  |  |  |
| Class 1 | 926 (5.93) | 207 (21.74) | 51 (5.36) |
| Class 2 | 7076 (45.32) | 1597 (22.51) | 559 (7.90) |
| Class 3 | 1592 (10.20) | 377 (23.68) | 145 (9.11) |
| Class 4 | 6019 (38.55) | 1398 (23.39) | 421 (6.99) |
